# *Enterococcus* Virulence and Resistant Traits Associated with Its Permanence in the Hospital Environment

**DOI:** 10.3390/antibiotics11070857

**Published:** 2022-06-26

**Authors:** Catarina Geraldes, Luís Tavares, Solange Gil, Manuela Oliveira

**Affiliations:** 1 Centre for Interdisciplinary Research in Animal Health (CIISA), Faculty of Veterinary Medicine, University of Lisbon, Av. da Universidade Técnica de Lisboa, 1300-477 Lisbon, Portugal; cgeraldes@fmv.ulisboa.pt (C.G.); ltavares@fmv.ulisboa.pt (L.T.); solange@fmv.ulisboa.pt (S.G.); 2 Laboratório Associado para Ciência Animal e Veterinária (AL4AnimalS), 1300-477 Lisbon, Portugal; 3 Biological Isolation and Containment Unit (BICU), Veterinary Hospital, Faculty of Veterinary Medicine, University of Lisbon, Av. Universidade Técnica, 1300-477 Lisbon, Portugal

**Keywords:** *Enterococcus*, virulence factors, antibiotic resistance, biocide resistance

## Abstract

*Enterococcus* are opportunistic pathogens that have been gaining importance in the clinical setting, especially in terms of hospital-acquired infections. This problem has mainly been associated with the fact that these bacteria are able to present intrinsic and extrinsic resistance to different classes of antibiotics, with a great deal of importance being attributed to vancomycin-resistant enterococci. However, other aspects, such as the expression of different virulence factors including biofilm-forming ability, and its capacity of trading genetic information, makes this bacterial genus more capable of surviving harsh environmental conditions. All these characteristics, associated with some reports of decreased susceptibility to some biocides, all described in this literary review, allow enterococci to present a longer survival ability in the hospital environment, consequently giving them more opportunities to disseminate in these settings and be responsible for difficult-to-treat infections.

## 1. Introduction

*Enterococcus* spp. are ubiquitous Gram-positive and facultative anaerobic bacteria, commensal of the intestinal tract of humans, as well as other mammals [1,2,3]. These microorganisms are largely characterized by their ability to tolerate high concentrations of salt (6.5% NaCl), but also a wide range of temperature (from 10 °C to 40 °C) and pH (from 4.4 to 9.6) values [1,2]. They are also able to hydrolyze esculin in the presence of high quantities of bile salts (40%) [2].

Initially portrayed as organisms of little clinical importance, enterococci, particularly *Enterococcus faecalis* and *Enterococcus faecium*, have been progressively associated with an increasing number of hospital-acquired infections (HAIs) in both human and veterinary medicine [3]. In fact, enterococci account for 6.1–17.5% of all isolates retrieved between 2010 and 2020 from European patients with these types of infections in human medicine [4]. Apart from these two species, other enterococcal species can also be isolated, such as *Enterococcus hirae*, *Enterococcus durans, Enterococcus gallinarium* and *Enterococcus casseliflavus*, not only in veterinary medicine [3], but also in human medicine [5,6,7], although they are not as commonly associated with HAIs and thus are less commonly studied. Enterococci are associated with a wide range of infections including urinary tract infections, bacteremia, endocarditis, wound infections (burn wounds or surgical incisions), abdomen and biliary tract infections, and infection of catheters and medical implants [8].

This infection-inducing capacity becomes especially critical when its degree of antibiotic resistance is considered. The intrinsic and extrinsic resistance traits associated with this genus allow it to be resistant to several antibiotics, including β-lactams, aminoglycosides and glycopeptides, rendering it difficult to combat these infections [1,3,4,8,9]. In fact, vancomycin-resistant *Enterococcus faecium* is considered by the World Health Organization (WHO) [10] as a high priority pathogen for which new antimicrobial therapies are needed.

This overly known resistance to antibiotics is not the only trait that makes *Enterococcus* a threat to human and animal health. They also present a great capacity of persisting in the environment [11,12], which might be associated with some reports of decreased susceptibility to certain biocides [13,14,15], especially in the presence of organic matter [16,17]. They also have a formidable biofilm-forming capacity [8], and they are known for their genome’s plasticity, which allows them to easily acquire, conserve and disseminate genetic traits among not only enterococci, but also other Gram-positive bacteria [8,18,19,20,21].

Bearing this in mind, this article presents a review of all the attributes that can justify the extended permanence of this genus in the hospital environment, which consequently leads them to be one of the most important bacteria in terms of HAIs.

## 2. Genetic Organization

It is said that enterococci capacity to acquire new genetic material is one of the most important traits that allows them to adapt to different environments [22]. This becomes even more evident when we consider that the complete genome sequence of the first vancomycin-resistant *E. faecalis* (V583) reported in the United States revealed that more than a quarter of its genome consisted of acquired DNA [23].

This adaptability capacity to different environments seems to have divided the *E. faecium* population into two different clades: clade A, more adapted to the hospital environment, and clade B, considered the community adapted clade [24,25,26,27,28,29,30]. The genome of clade A isolates is usually larger and contains a higher quantity of exogenous genetic material frequently associated to antibiotic resistance and the carbohydrate metabolism [22,25,31], and has been mostly associated to clonal complex (CC) 17 [24,32]. However, this division has been contested since some isolates do not group genetically in this clade [26,29]. Some authors also defend that clade A can be divided into two different sub-clades: clade A1, comprised of human clinical strains, and clade A2, usually an animal-associated group [28,32]; however, this separation branch has also been contested since recent studies found no proof for the existence of this sub-division [29,30].

Regarding *E. faecalis*, no such genetic division has been made since no correlation has been found between clonal structure and isolate origin [27,33,34]. However, some clonal complexes such as CC2, CC9 and CC87, termed high-risk enterococcal clonal complexes (HiRECCs) due to their multi-drug resistant (MDR) profile, have been more associated to the hospital environment and nosocomial infections [24,33,35].

## 3. Virulence

In the last few years, vancomycin-resistant *Enterococcus faecium* have been a rising cause of concern in terms of enterococcal infections. However, when it comes to virulence factors, *E. faecalis* has the leading role, which explains why it is still considered the primary species in terms of nosocomial infections. Although they are not a main issue in the *Enterococcus* genus, they still represent an advantageous feature in terms of environmental survival since some of these factors can be associated to biofilm formation and adherence to a variety of surfaces. Virulence factors can be divided into two distinct groups: those that are secreted, and those that are present in the surface of the bacterial cell [32]. In this article, we present the most important virulence factors included in both of these categories.

### 3.1. Secreted Virulence Factors

One of the first virulence factors described in enterococci was cytolysin, encoded by the genes *cylLL* and *cylLS* [36], and named due to its dual action, since it presents both a bactericidal and cytolytic activity [37,38,39]. Cytolysin seems to present some activity against red and white blood cells from mice [40], and also seems to contribute to endophthalmitis [41] and endocarditis [42]. Although a study by Jett et al. in 1992 [41] on a rabbit model indicated a possible association between cytolysin-producing enterococci and endophthalmitis, more recent studies do not seem to find any connection between the two [43,44].

Exclusively present in *E. faecium* isolates [31], secreted antigen A (SagA) is a stress-related protein [45] that supposedly also plays a role in cell growth, conceivably due to interactions with the cell wall metabolism [46]. It can additionally bind to a series of extracellular matrix proteins [47]. On the other hand, this protein has also been correlated to a better functioning intestinal barrier and enhanced tolerance against other enteric pathogens such as *Salmonella* Typhimurium and *Clostridium difficile*, possibly due to activation of the host innate immune system [48,49,50]. SagA has also been associated with biofilm formation, but only in relation to enterococci belonging to clade A1 [50].

Gelatinase, encoded by the *gelE* gene, is a matrix metalloproteinase, best studied in *E. faecalis* [8,32]. This protein is capable of hydrolyzing gelatin and collagen and not only seems to be able to interfere with complement-mediated immunity [51], but also seems to be an aid in the development of infectious endocarditis by *E. faecalis* [52]. GelE is co-transcribed along with SprE, a serine protease, by the FSR system [53,54], which also appears to regulate other types of proteins, some of them associated with biofilm formation [55]. GelE seems to reduce the incidence of Ace, another virulence factor present at the cell surface, possibly due to a GelE-dependent cleavage of the Ace protein, which reduces the enterococci ability to bind to collagen [56].

The *hyl_Efm_* genes encode a glucosyl hydrolase, and is predominantly present in clinical isolates, especially *E. faecium* CC17 [57,58]. Although, to the best of our knowledge, there are not many studies that indicate the potential of this protein as a virulence factor, it has been suggested that it could lead to the co-transference and dissemination of antibiotic resistance genes [59].

### 3.2. Cell Surface Virulence Factors

Cell surface components have been found to be important in a series of different bacterial defense mechanisms, including biofilm formation and protection against the host immune system [60,61].

In enterococci, these surface proteins are usually of the LPxTG-type (leucine, proline, X (any amino acid), threonine, and glycine), which include pili and microbial surface components recognizing adhesive matrix molecules (MSCRAMMs) [62].

The first ever described cell surface group of proteins included the aggregation substances (also known as AS or Agg), which depict a group of three adhesins: Asp1, Asc10 (sometimes also called PrgB) and Asa1. These proteins are encoded by three distinct conjugative plasmids, pPD1, pCF10 and pAD1, respectively, and are very similar in amino acid sequence [62]. These AS have been considered to be responsible for enterococcal adhesion to intestinal [63,64,65] and renal tubular cells [66]. As such, this is possibly associated with their ability to prompt systemic infections [62], since adhesion is usually the first step necessary for infection. This takes an even greater importance knowing that Asa1 adheres to extracellular matrix proteins, which also facilitates infection development [67]. Furthermore, AS have also been correlated to adhesion to immune system cells (especially Asa1 and Asc10) [68,69], and phagocytosis survival [69,70], as well as *vanA* (an operon associated to vancomycin resistance) co-transference in *E. faecalis* [71]. Asc10, which is encoded by the *prgB* gene, has also been associated to a higher virulence in infective endocarditis [72] and biofilm formation [73].

There are three main MSCRAMMs, which are a subfamily of bacterial adhesins that recognize and bind to extracellular matrix elements, described in enterococci: Ace (adhesion of collagen, from *E. faecalis*) [74], Acm (adhesion of collagen, from *E. faecium*) [75] and Scm (second collagen adhesion, from *E. faecium*) [76]. 

Ace was the first MSCRAMM identified in enterococci [74], followed by Acm [75], and lastly by Scm [76]. Although they all promote adhesion to collagen, Ace has also been characterized by its capacity to adhere to other components such as laminin [77] and dentin [78], while Scm also binds to fibrinogen [76]. Both Ace and Acm seem to play a role in the development of infectious endocarditis caused by enterococci [79,80,81]. Although all three are present in clinical and non-clinical isolates, Acm seems to be predominantly distributed among *E. faecium* clinical isolates, with a higher incidence among CC17, which could be one of the reasons this complex gained such importance as a nosocomial pathogen [82].

Pili are another relevant virulence factor that consist of multimeric fibres composed of pilin subunits that extend as long filaments from the cell surfaces. In enterococci, these fibres are encoded by an operon consisting of a collection of different genes, commonly called pili gene clusters (PGC).

The *E. faecalis* genome contains two distinct PGC: EBP (endocarditis and biofilm-associated pilus), composed of three distinct genes, *ebpA*, *ebpB* and *ebpC*; and BEE (biofilm enhancer in *Enterococcus*), composed of three pili-enconding genes, *bee-1*, *bee-2* and *bee-3*, and two sortase-like enzyme-encoding genes, *srt-1* and *srt-2* [83]. While EBP seems to be ubiquitously distributed among *E. faecalis* [84], the BEE PGC is much less frequently found [83]. Both these clusters seem to be important in biofilm formation [83,85].

On the other hand, *E. faecium* is considered to have four different gene clusters, which were identified as PGC1 to 4 [62,76,86]. PGC3, known as EMP (previously identified as EBP_fm_) is composed by three different genes, *empA*, *empB* and *empC* [87,88], and has been associated with biofilm formation and adherence to EMCs [88], as well as colonization of both the kidneys and the bladder in an experimental urinary tract infection model [87].

Esp is another cell surface protein, present in both *E. faecium* and *E. faecalis*, which seems to be predominantly present in clinical isolates [89,90]. This protein has been proven to be important in biofilm formation [91,92,93], possibly through an amyloid-based mechanism [94], but has also been implicated in infectious endocarditis [95] and urinary tract infection development [96]. Furthermore, a study published in 2009 by Meredith et al. [97] also indicated a possible relation between the presence of this protein and *E. faecium*’s susceptibility to β-lactams, which has also been corroborated by a more recent study [98].

Other cell surface proteins acting as potential virulence factors have also been described, such as PrpA [99], that binds to fibrinogen, fibronectin and platelets, and SgrA and EcbA, two adhesins that seem to be predominantly present in *Enterococcus* clinical isolates [100].

**Figure 1:**
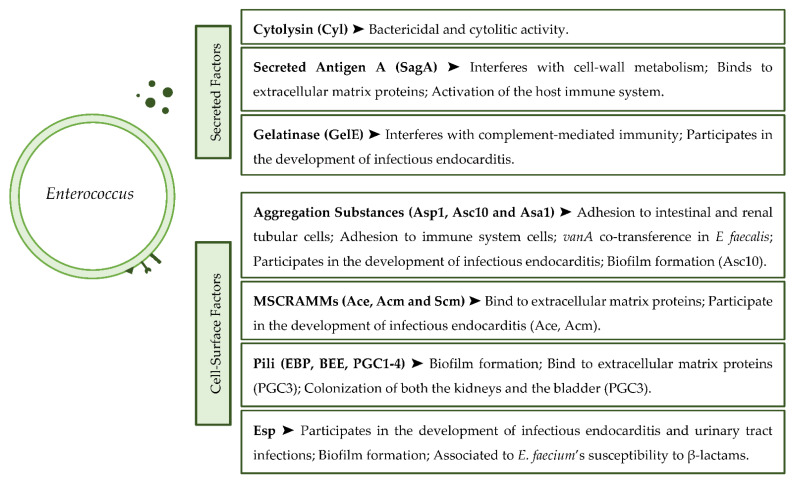
Summary diagram of the most relevant virulence factors present in enterococci.

The most important virulence factors presented by the *Enterococcus* genus can be seen summarized in Figure 1.

## 4. Antibiotic Resistance

### 4.1. β-Lactam Resistance

*Enterococcus* present a variable intrinsic resistance to the different classes of β-lactams; they usually present only a reduced susceptibility to penicillins, which justifies the fact that amoxicillin and ampicillin are still considered the first line of antibiotic defence against this group of bacteria in both animal [101] and human medicine [1,8,9], respectively. Carbapenems are slightly less efficient towards enterococci, and they are considered resistant to cephalosporins [1,3,8,9].

Bacterial D,D-transpeptidases, also known as penicillin binding proteins (PBPs), are responsible for the last step of cross-linkage in peptidoglycan formation, a complex structure responsible for the stability and rigidness of the bacterial cell wall. β-lactams have the capability of acting as substrates for these PBPs, effectively inhibiting them and, consequently, preventing cell wall formation [102].

In enterococci, intrinsic resistance is usually associated with the existence of low-affinity PBPs that do not allow binding of the antibiotic molecules as easily. The presence of these proteins has been associated with low-level resistance to penicillins and moderate to high-level resistance to cephalosporins [3,8]. PBPs are usually divided into high-molecular and low-molecular weight PBPs. From both these groups, high-molecular PBPs are the ones associated with β-lactam resistance in bacteria [103]. These transpeptidases can also be divided into two classes: class A that presents both transpeptidase and transglycosylase activity, and class B that presents only transpeptidase activity [104].

It has been known for a long time that *Enterococcus* strains have at least five PBPs [105], associated with six putative genes: three of those genes belong to Class A (*ponA*, *pbpF*, *pbpZ*), and three to Class B (*pbp5*, *pbpA*, *pbpB*) [103,104]. The chromosomally encoded *pbp5* gene, present in *E. faecium* strains, has been the gene most frequently associated to penicillin and cephalosporin resistance in *Enterococcus* [106]. Although this gene has also been identified in *E. faecalis* (*pbp4*), it has not been as associated to β-lactam resistance in this species as in *E. faecium* [104]. In fact, *E. faecalis* usually presents higher rates of susceptibility to β-lactams in comparative studies [107,108,109]. It has also been proven that the *pbp5* gene is a transferable element between *E. faecium* and is sometimes associated with the transference of vacomycin-resistance determinants [110,111,112].

A higher resistance to these antibiotics is usually associated with a higher expression of PBP5 proteins [113,114] or to mutations in the amino acid gene sequence that lead to alterations of this protein’s molecular structure [114,115,116]. When evaluating the relation between *pbp5* sequences of *E. faecium* and penicillin’s minimal inhibition concentration (MIC) presented by these isolates, a study by Galloway-Peña et al. [115], corroborated later by another study by Pietta et al. [116], concluded that isolates that presented a MIC ≤ 2 µg/mL and isolates that presented a MIC ≥ 16 µg/mL had a 5% difference in the *pbp5* sequence, which remounted to the presence of two distinct allelic forms: a more susceptible (PBP5-S) and a more resistant (PBP5-R) form. These authors also proposed the existence of a hybrid PBP5 (PBP-S/R) for the isolates that present a MIC ≈ 4 µg/mL, whose genomic sequence fell between the other two genes. These differences were also associated to different enterococci clades previously mentioned, with PBP5-R being more associated to Clade A1 and PBP5-S to Clade B [113,115,116]. However, both studies concluded that the determination of which amino acid changes were responsible for this decrease in susceptibility to β-lactams was difficult. The divergence seen in results described in other studies also reinforces this conclusion, although some changes such as an additional serine after amino acid position 466 (Ser-466) or amino acid substitutions (for instance the substitution of a methionine, Met-485, for an alanine or threonine (Met-485 → Ala/Thr), the substitution of a glutamic acid, Glu-629, for a valine, and also the substitution of an aspartic acid, Asp-496, for a lysine) have been more frequently associated to a high-level of penicillin resistance than others [114,115,117,118,119]. Although it has been proven that this PBP is responsible for some degree of resistance to penicillins, high-level resistance cannot be solely justified by the increased expression or mutations of the *pbp5* gene, which means that other factors should also play some kind of role in this adaptation [106,114].

On rare occasions, decreased susceptibility to β-lactams has been also associated with the production of β-lactamases, with Murray reporting the first *E. faecalis* β-lactamase producer in 1983 [120] and Coudron et al. [121] reporting the first *E. faecium* β-lactamase producer in 1992. Since then, reports on β-lactamase producing enterococci have been scarce and more frequently associated with *E. faecalis* [1,122]. β-lactamases act through β-lactam ring hydrolysis, originating a molecule incapable of binding to the PBP [102]. Even though *Enterococcus* and *Staphylococcus* present a very similar sequenced operon, which indicates the possibility that these genes were transferred from one genus to the other [122], the *blaZ* gene in enterococci has a low-level constitutive expression, which means that it occurs independently of the presence of β-lactams [104]. This means that an in vitro susceptibility may not be equivalent to an in vivo one, since infections by these bacteria equivalate to higher concentrations of β-lactamases than the ones seen in vitro [3,104]. However, considering that the isolation frequency of these bacteria is reportedly low and also that these β-lactamases seem to be susceptible to β-lactamases inhibitors, such as sulbactam [104,122], resistance to β-lactams through this mechanism is not as concerning as through PBPs.

### 4.2. Aminoglycosides

Aminoglycosides act through disruption of mRNA decoding by binding to the 16S rRNA of the 30S ribosomal subunit [9]. This means that in order to exert their action, they must first pass through the bacterial cell wall.

Enterococci are known to present an intrinsic resistance to aminoglycosides due to two distinct factors: poor uptake of this antibiotic group through the cell wall, and through modification of the antibiotic molecule, reducing its affinity to their target [3,8,9,104].

In aminoglycosides, modification of the antibiotic molecule occurs due to a group of enzymes that can be divided into three different categories: the acetyl-coenzyme A-dependent amino-glycoside acetyltransferases (AACs), that act through acetylation of the amino groups; the ATP-dependent nucleotidyltransferases (ANTs), that act through adenylation of the hydroxyl group; and the ATP/GTP-dependent phosphotransferases (APHs), that act through phosphorylation of the hydroxyl group [9,123]. These molecules are also divided into sub-groups according to which position is altered (3′, 6′, 2″,3″, 4″, etc.), and according to the resistance profile presented (I, II, III, etc.). Letters (a, b, c, d, e, etc.) were added at the end in order to distinguish each specific protein [1,123,124]. 

In enterococci, there have been three similar transferases described as being chromosomally encoded and that confer low- to moderate-level resistance to aminoglycosides: AAC(6′)-Ii in *E. faecium* [125,126,127]; AAC(6′)-Id in *E. durans*; and AAC(6′)-Ih in *E. hirae* [127], conferring resistance to kanamycin and tobramycin [126]. Some aminoglycoside-modifying enzymes have also been associated to acquired resistance, by the obtention of genes encoded in transposons and conjugative plasmids. All these enzymes are presented in Table 1, along with the type of resistance they confer.

From all the presented enzymes, AAC-6′-Ie-APH-2 seems to be the most frequently associated with high-level resistance to gentamicin, and ANT(6′)-Ia to high-level resistance to streptomycin [138,139,140,141,142,143,144]. It is important to note, however, that a discordance between phenotype and genotype can occur when it comes to these enzymes, which means that not all bacteria that present a resistance gene for aminoglycosides presents a resistant phenotype [145]. This means that phenotype determination is always important in order to determine resistance to these antibiotics.

Enterococci with the ability to produce these enzymes, conferring high-level resistance to both gentamicin and streptomycin which are the go-to aminoglycosides to control enterococcal infection, are also usually resistant to the synergism between these antibiotics and cell wall active agents such as β-lactams [1,8,9].

Another noteworthy reference is the fact that there is a possibility that, in polymicrobial infections, modifying enzyme-producing enterococci could possibly shield other bacteria from aminoglycoside action, increasing the MIC values necessary to inhibit bacterial multiplication [146].

Intrinsic resistance to tobramycin and kanamycin, in both *E. faecium* and *E. faecalis*, is associated to a rRNA methyltransferase, EfmM, that acts through alteration of the ribosomal target-site [147]. 

High-level resistance to streptomycin has also been associated to punctual ribosome mutations [3,8,148].

Additionally, EfrAB, an ABC-transporter efflux pump, also seems to be responsible for the reduction of gentamicin’s MIC levels in enterococci [149].

### 4.3. Glycopeptides

It is known that glycopeptides, such as vancomycin and teicoplanin, act by binding to the peptidoglycan pentapeptide precursor, more specifically to the D-alanine-D-alanine (D-Ala-D-Ala) terminus, which blocks the last crosslinking step of peptidoglycan formation, consequently preventing cell wall formation [3,150]. In enterococci, the main mechanism of resistance to these antibiotics is the alteration of the terminus molecule, D-Ala-D-Ala, which corresponds to the glycopeptides target-site, thus reducing the affinity of these antibiotics to these targets [3,9,150].

The operons responsible for this modification are normally divided into two groups, according to the alteration they engender: *vanA*, *vanB*, *vanD* and *vanM* operons, which lead to the creation of a D-alanine-D-lactate (D-Ala-D-Lac) terminus associated to both vancomycin and teicoplanin moderate- to high-level resistance [150,151,152,153,154]; and the *vanC*, *vanE*, *vanG*, *vanL* and *vanN* operons responsible for the creation of a D-alanine-D-serine (D-Ala-D-Ser) terminus and associated only to low-level vancomycin resistance [150,151,153,154,155,156,157,158].

*E. faecium* is the most common species associated to glycopeptide resistance and *vanA* is the most described operon, with *vanB* coming in second; both are present in transposons that are either chromosomally encoded or transferable through plasmids, namely Tn1546 for *vanA*, and Tn1547 or Tn1549 for *vanB* [1,3,8,104,150,151].

**Figure 2:**
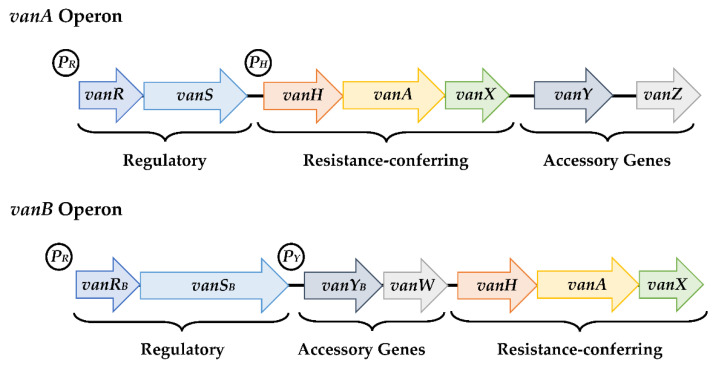
Representation of the organization of the *vanA* and *vanB* operons. PR, PH, PY: promoting regions. Adapted from Refs. [8,151,153].

The *vanA* operon is composed by seven different genes: *vanR*, *vanS*, *vanH*, *vanA*, *vanX*, *vanY* and *vanZ*, all working in sequence in order to develop glycopeptide resistance (Figure 2) [1,8,9,104,153].

Regulation of these genes is performed by the VanR-VanS system. In the absence of glycopeptides, the sensor kinase, VanS, acts as an inhibitor to the response regulator, VanR [8,157]. However, when glycopeptides are present in the environment, VanS activates the *vanR* gene through phosphorylation [1,9]. This gene subsequently activates a promoting region responsible for the transcription of three other genes: *vanH*, *vanA* and *vanX*. It also increases the regulation of the VanR-VanS system [8,159]. Moreover, *vanH* originates a dehydrogenase that reduces pyruvate to D-Lac that, along with the ligase produced by *vanA*, promotes the formation of the D-Ala-D-Lac terminus [9,104,153]. The *vanX* gene is responsible for the formation of a dipeptidase and *vanY* encodes a carboxypeptidase, both responsible for cleaving the remaining D-Ala-D-Ala forms. While VanX cleaves the free D-Ala-D-Ala terminus before its connection to the peptidoglycan precursors, VanY cleaves this same terminus after the pentapeptides’ formation [9,104,153,160,161]. On another end, *vanZ* has a still unknown mechanism of action that is somehow related to teicoplanin resistance [6,162].

The *vanB* operon is very similar to the *vanA* (Figure 1), with only two major differences: first in the regulatory system, which is encoded by *vanRB* and *vanSB*; and second in the absence of the *vanZ* gene, which is exchanged for the *vanW* gene, and the reason why enterococci presenting resistance to glycopeptides due to this operon are susceptible to teicoplanin [3,153].

Other operons have been described in enterococci, such as *vanC*, which is associated with intrinsic resistance in *E. gallinarium* (*vanC1*), *E. casseliflavus* (*vanC2*) and *E. flavescens* (*vanC3*) [3,153,163,164]. Other operons are of scarcer frequency in enterococci [8], and have been described extensively elsewhere [151,153,165].

### 4.4. Fluoroquinolones

DNA replication is essential in order for cell division to occur. This replication is dependent of the activity of two enzymes: DNA gyrase, responsible for the negative supercoiling of DNA and essential for transcription occurrence; and topoisomerase IV, responsible for the disentanglement of the newly replicated DNA and segregation of the daughter chromosomes [104,166,167]. Both of these enzymes are heterotetramers with two different subunits—GyrA2GyrB2 for DNA gyrase and ParC2ParE2 for topoisomerase IV—and are formed due to the expression of two different groups of genes, *gyrA/gyrB* and *parC/parE*, respectively [166,167].

Quinolones act by binding and inhibiting both of these enzymes, which impedes DNA replication and consequently cell division [9,166]. Initial reports associated enterococci resistance to quinolones mainly to mutations in the *gyrA* gene [168,169,170]. In 1998, Kanematsu et al. [171] also corroborated the fact that these resistances arise from mutations in the *gyrA* gene, but additionally indicated that they were also associated to mutations in the *parC* gene. Since then, numerous studies have associated high-level quinolone resistance in *E. faecium* and *E. faecalis* to mutations in both of these genes [172,173,174,175,176]. These mutations alter the affinity of these enzymes to quinolones, leading to an impediment in the formation of the quinolone-enzyme-DNA complexes, responsible for the inhibition of cell division [104,166]. 

Additionally, Kanematsu et al. [171] also pointed out the fact that some isolates presenting low-level resistance to quinolones only present mutations in the *parC* gene, which could be an indication that points to topoisomerase IV being the primary target of quinolones in enterococci. This seems to be the general case in Gram-positive bacteria, contrary to what happens in Gram-negative bacteria, in which DNA gyrase is considered generally the primary target [8,166,167,171]. This is also corroborated by the fact that Gram-positive DNA gyrase appears to be less susceptible to quinolones when compared to the one of Gram-negative bacteria [166]. However, Oyamada et al. [177] also indicated that the primary target could change according to the quinolone used. 

On the other hand, mutations in the *gyrB* and *parE* have been very infrequent in these bacteria [178].

Low-level resistance to quinolones in enterococci has also been associated to the activity of efflux pumps [178], mainly EmeA, a MDR efflux pump homolog to *Staphylococcus aureus* NorA [179].

Finally, in 2007, Arsène and Leclercq [167] also found, in *E. faecalis*, a protein homologue to QnrA, which they named Qnr *_E. faecalis_*. Similarly to QnrA, which is responsible for the protection of DNA gyrase and topoisomerase IV from fluoroquinolone inhibition, Qnr *_E. faecalis_* seems to be associated to resistance to ciprofloxacin through DNA gyrase protection [167,180,181,182].

### 4.5. Tetracyclines

Tetracyclines act through inhibition of protein synthesis by binding to the ribosome and preventing tRNA linking, causing a bacteriostatic effect upon the cell [104].

Resistance to this group of antibiotics has been extensively described in a great number of bacteria and, in enterococci, it has mostly been related either to two genes associated to efflux pumps, *tet(K)* and *tet(L)*, or to three genes associated to ribosomal protection, *tet(M)*, *tet(O)* and *tet(S)* [3]. Of all five genes, *tet(M)* seems to be the most frequently isolated from enterococci strains from both human and animal origin, with *tet(L)* coming in close second [142,173,183,184,185,186]. 

Transference of these genes through the Tn916/Tn1545 transposon family has also been associated to the co-transference of the gene *erm(B)*, associated with macrolide, lincosamide and streptogramin B (MLSB) resistance [3,183,184,187].

Recently, resistance to tetracyclines in various Gram-positive bacteria including enterococci has also been associated to an ATP-binding cassette (ABC)-F ribosomal protection protein, encoded by the *poxtA* gene. This gene can either be present in the chromosome or be encoded in a transferable plasmid, and is also associated to co-resistance to phenicol and oxazolidinones [188,189,190,191,192].

### 4.6. Oxazolidinones

Oxazolidinones, especially linezolid, are one of the antibiotics used against vancomycin resistant enterococci (VRE) [193,194,195,196]. Although the percentage of resistance seen in enterococci to this antibiotic is still low (<1%), it has been progressively increasing in the last few years [157,192,197,198,199,200,201,202].

Linezolid acts by binding to the 23s rRNA blocking tRNA docking, and thereby preventing protein translation [104,203]. 

The most common mechanism of resistance to this antibiotic, especially in *E. faecium*, consists of point mutations in the 23S rDNA, with the most frequent being the G2576T (*E. coli* numbering) [198,201,204]. While *E. faecium* has six genes encoding for this rRNA, *E. faecalis* has four, and the number of genes that suffer from this mutation has been associated to the degree of resistance presented by these bacteria [8,104,205]. 

Other mutations, including in ribosomal stabilization proteins L3 (*rplC*), L4 (*rplD*) and L22 (*rplV*), have also been described; however, they seem to be very rare in enterococci [204].

Acquisition of linezolid resistance is also associated to Cfr and Cfr-like methylases (in *Enterococcus* case *cfr(B)* and *cfr(D)*) [192,203,204,206,207,208,209,210,211], ABC-F proteins *optrA* [197,199,201,212,213] and the previously mentioned PoxtA [192,200,204]. The *optrA* gene seems to be the most frequent mechanism of resistance to linezolid in *E. faecalis* [204,212].

Cfr proteins confer resistance through post-transcriptional methylations of the 23S rRNA, which alter susceptibility not only to oxazolidinones, but also to phenicols, lincosamides, pleuromutilins and streptogramin A (PhLOPSA phenotype) [207]. However, the presence of these genes in enterococci does not always equal a resistance phenotype to linezolid [209,211].

OptrA and PoxtA lead to decreased susceptibility to phenicols and oxazolidinones, with PoxtA also conferring resistance to tetracyclines [188,189,197]. While *optrA* and *poxtA* are both usually acquired through plasmids, the *poxtrA* gene can also be chromosome encoded [192].

## 5. Biocide Tolerance

Biocides have been used for a long time with the intent of reducing the quantity of microorganisms present in different surfaces, and are helpful in the prevention of the growing quantity of multi-resistant organisms, the spread of infections and, consequently, the amount of HAIs occurring in today’s practice. Regulation (EU) n^o^ 528/2012 of the European Parliament and the Council of 22 May 2012 defines “biocidal product” as a compound that contains in its composition (or that leads to the formation of) one or more active substances, utilized with the intent of “destroying, deterring or rendering harmless” microorganisms (by other means besides physical or mechanical ones), in order to attenuate or eliminate any detrimental action these agents may have towards host health. These compounds are usually divided into four categories: antiseptics, sterilants, disinfectants and preservatives; however, several compounds can fit into more than one category [214].

Although the term “resistance” is widely used in the “antibiotic world”, the same cannot be said for biocides. In relation to this group of compounds, terms such as “reduced susceptibility” or “tolerance” are more frequently used since while they are associated with increases in the minimal inhibitory/bactericidal concentrations (MICs/MBCs) needed to either inhibit or kill a certain bacterium, they also imply that in-use concentrations are still effective against these microorganisms [215]. This definition, however, becomes a little more unclear when we contemplate the lack of standardized laboratory methods that can be used to determine biocide susceptibility, and MIC/MBC breakpoints that define the line between biocide tolerance and susceptibility [216].

There are a variety of factors that can affect a biocide’s efficiency and lead to bacterial tolerance. These factors can be related to the biocide itself, such as its concentration, pH and formulation; to the treatment conditions in which these compounds are applied, such as the temperature, presence of organic matter and contact time; or to the targeted microorganisms, due to differences in the cell wall, the presence of efflux mechanisms or enzymatic degradation. The presence of organisms organized in the biofilm form are also associated to a higher biocide tolerance [217]. When considering biocide tolerance, concentration is usually considered the most important factor [215,217]; nevertheless, all other factors should be considered in order to achieve an optimal biocidal efficiency and reduce possible decreases in susceptibility.

In enterococci, such as in other bacteria, increases in tolerance to biocides could be associated to protective stress-associated mechanisms triggered when in the presence of sub-lethal concentrations of these compounds, either due to a wrongful application or to biocidal residues left in the environment after usage [16].

Small RNAs (sRNAs), typically composed of noncoding transcripts between 50 and 600 nucleotides, are usually produced under specific environmental conditions [218]. These sRNAs, still being studied in both *E. faecalis* [219,220] and *E. faecium* [218], have been thought to act as a regulatory system of a network of genes [219], activated when under antibiotic [218] or biocide [221] stress, possibly leading to an adaptation to both of these antimicrobials and consequent decreases in susceptibility.

Phosphotransferase systems (PTS), responsible for the transportation and phosphorylation of sugars used by bacteria to produce energy, have also been proven to be important in *E. faecalis* survival to a variety stress conditions [222]. This system has been indicated by Pidot et al. [14] as a possible explanation for an increase in alcohol tolerance in *E. faecium*.

On the other hand, the two-component system ChtRS, that also reacts to environmental alterations and is coded by the *chts* and *chtr* genes that putatively encode a histidine kinase and a response regulator, has been associated to chlorhexidine tolerance in *E. faecium* [12].

Efflux systems, such as QacA/B and EfrAB, have equally been associated to biocide tolerance in enterococci, especially to chlorhexidine [149,223].

Moreover, it seems valuable to indicate that different enterococci species seem to present different tolerance levels to different biocides, with the most tolerant species variating according to the biocide tested [224]. This means that when studying the efficacy of a biocide against *Enterocccus* or bacterial tolerance in this genus, different species should be tested.

There has also been a great debate on whether there is a possible association between biocides and antibiotics resistance, with some studies indicating this possibility [14,225], while other studies conclude that a link between the two does not exist [217,223]. What seems to be mostly accepted is that the existence of this association probably depends on the antibiotic and biocide tested and the resistance mechanisms associated. However, the application of sub-lethal concentrations of biocides could possibly co-select antimicrobial-resistant enterococci [226].

This resilience and capability of adaptation to stressful conditions presented by the *Enterococcus* genus is very much a concern, and it goes beyond antibiotics and biocides, with reports indicating possible decreases of susceptibility even to venoms [227].

Table 2 presents a summary of the main mechanisms of resistance and decreased susceptible to antibiotics and biocides, presented by the *Enterococcus* genus.

## 6. COVID-19 and *Enterococcus*

One of the most menacing HAIs of the last few years has been the severe acute respiratory syndrome coronavirus 2 (SARS-CoV-2) infection. This infection, like most viral infections, is known to lead to patient immunosuppression, and consequently give-way to secondary bacterial infections caused by commensal opportunistic pathogens [228].

However, among all the opportunistic pathogens, enterococci seem to strive in the presence of SARS-CoV-2 infected patients, especially when it comes to bloodstream infections (BSI) [229,230]. This relation is still not completely clear [229], with some studies indicating that these enterococci could be of nosocomial origin [231], while others indicate that they could originate from the individual himself, due to disruption of the intestinal barrier caused by viral multiplication and a consequent increase of enterococcal concentration in the gut [232].

Considering all these factors, it is also important to note that during the COVID-19 pandemic there was an increase in antimicrobial usage in order to fight these secondary infections, which means that a corresponding increase in bacterial resistance to these compounds is also to be expected [229]. This has been reflected in the enterococcal population with reports of increments in highly resistant strains of *Enterococcus* [233], including VRE [234].

Although the SARS-CoV-2 infection’s consequences and association with enterococcal infection have been, and are still being, thoroughly studied in human medicine, this has not been the case in veterinary medicine. It has been proven that both cats and dogs can become infected [235]; however, to the best of our knowledge, the consequences of these infections have not been studied in relation to the enterococcal population. Nevertheless, we hypothesize that these consequences could be similar to those seen in human medicine and should be carefully studied in order to understand what kind of impact they could possibly have in combating enterococcal infections.

This means that although enterococci have been gaining relevance as an infection-causing pathogen, especially due to all the factors presented in this review, they become even more a point of concern when considering their increased relevance due to the COVID-19 pandemic.

## 7. Conclusions

Enterococci seem to have developed a variety of mechanisms that make them more apt at survival in the hospital environment. This capacity seems to be a result of the combination of diverse factors.

Although this genus is not known for its variability of virulence factors, all those described in this review seem to contribute to the development of infection and also to the increased resilience in the presence of adverse conditions, especially due to cell surface proteins, such as Esp, that aid in biofilm formation, structures known for their resistance not only to antibiotics but also to biocides.

On the other hand, the intrinsic resistance these bacteria present to a multitude of antibiotics, associated with their genome plasticity and consequent capability of acquiring new genetic elements connected to this same resistance, makes them a concern in terms of antibiotic therapy, especially in terms of vancomycin-resistant enterococci.

Finally, although representing a field that requires further studying, their multiplicity of stress survival-related mechanisms could make them less susceptible to biocides and consequently able to last in the environment.

All of the characteristics described in this article associated with a possible potentiation by COVID-19 of this genus, which make them a number one priority in terms of vigilance and infection control.

## Figures and Tables

**Table 1:** Representation of all of the aminoglycoside-modifying enzymes that have been described in the *Enterococcus* genus, along with the type of resistance presented and the respective reference.

Enzyme		Type of Resistance Conferred		Reference
AACs	AAC(6′)-Ii	Intrinsic	Low- to moderate-level resistance to tobramycin and kanamycin	[125,126]
	AAC(6′)-Id			[127]
	AAC(6′)-Ih			[127]
APHs	APH(3′)-IIIa	Extrinsic	Low-level resistance to kanamycin and amikacin	[128]
	APH(2″)-Ib	Extrinsic	High-level resistance to gentamicin	[129]
	APH(2″)-Ic			[130]
	APH(2″)-Id			[131]
	APH(2″)-Ie			[132]
ANTs	ANT(6′)-Ia	Extrinsic	High-level resistance to streptomycin	[3,133]
	ANT(3″)-Ia or ANT(3″)(9)			[3]
	ANT(4″)-Ia			[134]
Bifunctional Enzyme (AAC + APH)	AAC-6′-Ie-APH-2	Extrinsic	High-level resistance to gentamicin	[135,136,137]

**Table 2:** Summary table of the main mechanisms of resistance and decreased susceptibility to antibiotics and biocides, presented by the *Enterococcus* genus.

Antibiotics			
Group of Antibiotics	Resistance Type	Mechanism of Resistance	Associated Genes
β-Lactam	Intrinsic	Low affinity PBPs that do not allow for easy antibiotic binding	*pbp5*/*pbp4*
	Acquired	Mutations that lead to alteration in PBPs’ molecular structure and cause an even lower affinity	-
Aminoglycosides	Intrinsic	Poor antibiotic uptake trough the cell wall	-
	Intrinsic	Modification of the antibiotic molecule	*aac*
	Acquired	Modification of the antibiotic molecule	*aph*, *ant*, *aac-aph*
	Intrinsic	Target-site modification through rRNA methyltransferase	*efmM*
	Acquired	Target-site modification through point mutations	*-*
	-	Efflux of the antibiotic	*efrAB*
Glycopeptides	Intrinsic/Acquired	Target-site modification	*van* operons
Fluoroquinolones	Acquired	Target-site modification through gene mutation	*gyrA, pacC*
	-	Efflux of the antibiotic	*emeA*
	-	Target-site protection	*qnr_E.faecalis_*
Tetracyclines	-	Efflux of the antibiotic	*tet(K), tet(L)*
	-	Target-site protection	*tet(M), tet(O), tet(S)*
	Intrinsic/Acquired	Target-site protection	*poxtA*
Oxazolidinones	Acquired	Target-site modification through point mutations	*-*
	Intrinsic/Acquired	Target-site protection	*poxtA*
	Acquired	Target-site protection	*optrA*
**Biocides**			
sRNAs	Bacterial survival in stressful environmental conditions such as in the presence of biocides		
PTS Systems			
ChtRS			
Efflux Pumps	QacA/B and EfrAB—efflux of different biocides		

## Data Availability

Not applicable.
